# Emergence and endemicity of arboviruses in Haiti: a review

**DOI:** 10.1016/j.ijregi.2026.100964

**Published:** 2026-07-26

**Authors:** Rigan Louis, Sk Nazmus Sakib, Z. Hugh Fan, Leslie Ann Parker, John A Lednicky, John Glenn Morris, Valery Madsen Beau De Rochars

**Affiliations:** 1 Emerging Pathogens Institute, University of Florida, Gainesville, FL; 2 College of Nursing, University of Florida, Gainesville, FL; 3 Department of Mechanical and Aerospace Engineering and Department of Biomedical Engineering, College of Engineering, University of Florida, Gainesville, FL; 4 Department of Environmental and Global Health, College of Public Health and Health Professions, University of Florida, Gainesville, FL; 5 Department of Health Services Research, Management, and Policy, College of Public Health and Health Professions, University of Florida, Gainesville, FL

**Keywords:** Arbovirus, Haiti, Review, Vector-borne diseases

## Abstract

**Objectives:**

The increase in geographical spread of arboviruses and the incidence of arboviral diseases of humans is a rising public health concern, particularly in the context of ongoing population movements, climate change, and challenges to public health surveillance systems in countries where many of these viruses are endemic. We present here a scoping review and a synopsis of the existing literature on arboviruses that have been reported in Haiti, identify existing challenges in public health surveillance, and discuss the implications for Haiti, the Caribbean, and the Americas.

**Methods:**

A scoping review of publications reporting arboviral infections among Haitians and returning travelers from Haiti, non-human vertebrates, or arthropod vectors was conducted following the PRISMA guidelines. Titles and abstracts screening, and full text reviews were performed independently by two reviewers.

**Results:**

A total of 556 articles were retrieved from 6 different databases, only 36 satisfied the inclusion criteria for this scoping review. At least 12 arboviruses associated with human diseases, including 11 mosquito-borne and one biting midge-borne, are or have been present in Haiti.

**Conclusion:**

Genetic data, where available, is consistent with ongoing movement of arboviruses within the Caribbean and South America. Where reliable, advance molecular approaches have been used, new and unexpected arboviruses have been identified, highlighting the importance of such diagnostic approaches for enhancing public health surveillance for endemic and new pathogens in Haiti.

## Highlights

• Twelve arboviruses associated with human diseases are or have been present in Haiti.
• These viruses belong to three genera: *Orthoflavivirus, Alphavirus*, and *Orthobunyavirus*.
• Existing evidence supports the movement of these viruses across the Caribbean.

## Introduction

The emergence of various vector-borne diseases (VBDs) in new geographical areas over the last few decades is a paradigmatic illustration of the impact of human globalization, urbanization and habitat destruction, and global warming on the transmission of emerging and new pathogens. VBDs account for more than 17% of all infectious diseases in some parts of the world and are responsible for an estimated 700,000 deaths annually, with disproportionate impacts in low- and middle-income countries [1]. Arthropod-borne viral diseases (arboviral diseases) are among the predominant VBDs [1]. Arboviruses are obligate parasites that are maintained through transmission between and within susceptible vertebrate hosts and hematophagous arthropods, including mosquitoes, ticks, sandflies, and biting midges [2]. Most arboviruses of public health importance belong to the Flaviviridae, the Togaviridae, and the Peribunyaviridae families, among which about 130 species are known to cause human diseases [3]. The majority of arboviral infections in humans are asymptomatic; only a few infected individuals experience clinical manifestations, which can range from mild febrile illnesses to hemorrhagic complications and severe encephalitis [3].

The Republic of Haiti covers an area of 27,750 km^2^ on the island of Hispaniola, which it shares with the Dominican Republic [4]. Haiti remains one of the poorest and least stable countries in the Western Hemisphere, with recurrent disease outbreaks, gang violence, constant political turmoil, and food scarcity that force mass family migration to neighboring countries [5]. With a favorable climate for arboviral vectors, limited waste management and drainage infrastructure, densely populated communities, and a vulnerable public health system, Haiti offers all the necessary conditions to sustain arboviral endemicity with cyclic outbreaks [4]. Ongoing conflict, persistent internal displacement, and mass immigration, coupled with recurrent natural disasters, may have direct and indirect effects on the transmission of infectious diseases within Haiti and in its neighboring nations [6].

The emergence of Chikungunya virus (CHIKV) and Zika virus (ZIKV) in the Western world over the last 13 years has bolstered research activities on arboviruses globally, including in Haiti, where such efforts have resulted in the identification of new arboviral species that were not known to circulate there [7]. Our group has been involved in research on arboviruses in Haiti for over 15 years. Building on this experience, we present here a scoping review and a synopsis of the existing literature on arboviruses that have been reported in Haiti, identify existing challenges in public health surveillance, and discuss the implications for Haiti, the Caribbean, and the Americas.

## Methods

This scoping review follows the Preferred Reporting Items for Systematic Reviews and Meta-Analyses extension for Scoping Reviews Checklist (Supplementary Table S1).

### Eligibility criteria for study inclusion

Peer-reviewed observational studies reporting arboviral infections in Haiti among Haitians, travelers returning from Haiti, non-human vertebrates, or arthropod vectors were considered for inclusion. To capture the full scope of the existing literature, no restrictions on publication date were applied. Studies published in languages other than English or French (Haiti’s official language) without an English or French translation, and review articles, were excluded.

### Information sources and search strategy

A comprehensive search strategy was developed using a combination of keywords and Medical Subject Headings terms, representing variations of different arbovirus species (Supplementary Table S2). Subsequently, the following databases were searched in August 2025 to retrieve eligible articles: MEDLINE/PubMed, Web of Science, Scopus, EMBASE, LILACS/VHL, and CINAHL. The references of all articles were carefully reviewed to identify additional eligible studies.

### Selection of information and data charting process

Retrieved articles from all databases were uploaded to Covidence [8]. After titles duplicated across multiple databases were removed, titles and abstracts were screened, and full-text reviews were performed independently by two reviewers. In case of any disagreement, consensus was sought through consultation with a third reviewer.

## Results

### Study selection

**Figure 1:**
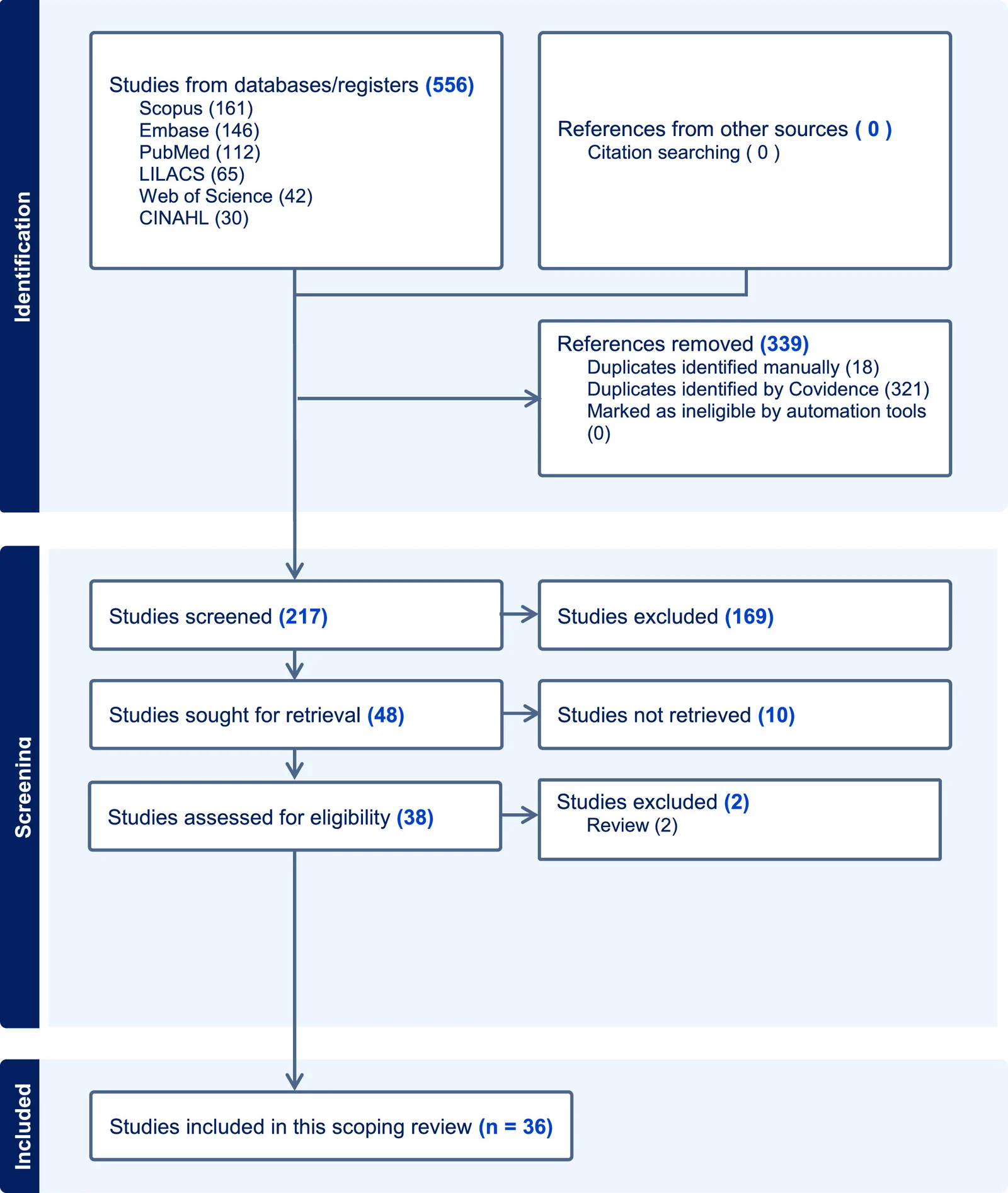
Flow chart depicting the screening process.

A total of 556 published articles, all in English, were retrieved from the six databases and were uploaded to Covidence. Subsequently, 339 duplicates were identified and removed (321 by Covidence and 18 manually). Title and abstract screening, followed by full-text review, identified 181 articles that did not satisfy the inclusion criteria, including 10 for which the full text was not retrievable. The remaining 36 articles were eligible for this review. Detailed information on the study selection process is presented in Figure 1.

### Characteristics of the included studies

**Figure 2:**
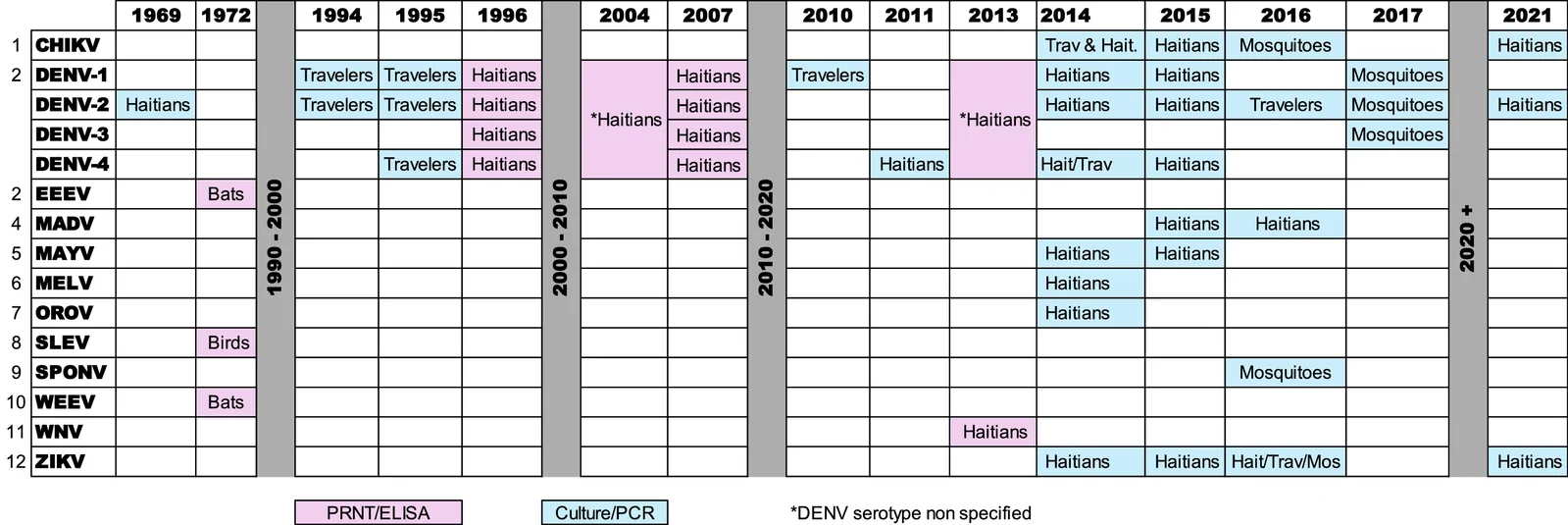
Arbovirus circulation in Haiti and timeline (1969-2021). Data collected from studies published on arboviruses in Haiti between 1976 and 2025 provide molecular and serological evidence supporting the circulation of 12 distinct arboviruses in the country. Viruses are grouped based on the year when the biological samples were collected.

Figure 2 and Supplementary Table S3 provide comprehensive information about the studies, including the authors, publication years, and diagnostic techniques. The studies were published between 1976 and 2025, with the majority (69.4%) published between 2010 and 2020. Notably, half of the studies were conducted in the Ouest Department, which includes Port-au-Prince, Haiti’s capital and largest city. In summary, the 36 studies presented herein provide evidence supporting the presence and potential circulation of at least 12 distinct arboviruses in Haiti. Eight of these viruses were confirmed by culture or polymerase chain reaction, while the rest were identified through serological assays. Eight viruses were identified in human clinical samples, with three of these viruses also being detected in mosquito vectors. Additionally, one virus was exclusively identified in mosquitoes. Neutralizing antibodies against the remaining three viruses were detected in non-human vertebrate hosts, including birds and bats. Thirteen studies reported arboviral infections among travelers returning from Haiti or people of Haitian origin living outside Haiti, with cases reported in Italy (n = 4), the United States (n = 4), among United Nations personnel (n = 3), France (n = 1), and Spain (n = 1); the remaining studies were cross-sectional or longitudinal sentinel surveillance studies conducted in Haiti.

### Synthesis of findings

The 12 viruses belong to three different genera, namely *Alphavirus, Orthobunyavirus*, and *Orthoflavivirus*, from the Togaviridae, Peribunyaviridae, and Flaviviridae families, respectively.

#### Orthoflaviviruses

The genus *Orthoflavivirus* contains approximately 53 species, the majority of which are arthropod-borne viruses transmitted by mosquito and tick vectors, and they primarily infect mammals and birds [9]. This review identified four *Orthoflavivirus* species that have been reported to circulate in Haiti, namely Dengue virus serotypes 1 to 4 (DENV-1, DENV-2, DENV-3, and DENV-4), St. Louis encephalitis virus (SLEV), West Nile virus (WNV), and ZIKV. A fifth flavivirus related to ZIKV has been identified in Haiti: Spondweni virus (SPOV) [10].

##### DENV

DENV was the most frequently reported arbovirus across the 36 articles, with persistent activity in Haiti over the last 50 years (Figure 2). All serotypes have been reported in Haiti among Haitian residents and returning travelers from Haiti, with the exception of DENV-3, for which no data were found from travelers (Figure 2). In addition, DENV serotypes 1, 2, and 3 were isolated from *Aedes aegypti* collected between March and June 2017 [11].

An increase in arbovirus surveillance associated with the emergence of CHIKV in the Americas in 2013 resulted in the additional collection of data on DENV in Haiti [11,12]. In 2021, during a surveillance study of acute febrile illnesses (AFI) in children residing along the border of Haiti and the Dominican Republic, a previously unrecognized outbreak of DENV-2 involving over 60% of study participants was identified [13]. Clinical findings included fever, abdominal pain, retro-orbital pain, rash, and arthralgias [13]. In contrast to findings in other dengue-endemic regions, none of the studies reported any instance of dengue hemorrhagic fever (DHF) or dengue shock syndrome (DSS), consistent with the perception among physicians and researchers that DHF and DSS are rare or do not occur in Haiti [13].

##### SLEV

Most cases of SLEV infection are asymptomatic, although in rare cases the virus affects the brain and causes encephalitis or meningitis [14]. The initial report of SLEV isolation was from a green heron in Haiti in 1955; neutralizing antibodies to SLEV were subsequently found in three non-migratory bird species (great blue heron, barn owl, and village weaver) during a serosurvey in 1972 [15].

##### WNV

Introduction of WNV into North America in 1999 is well documented, and the virus is currently the leading cause of mosquito-borne disease in the US [16]. Although WNV can cause severe and sometimes fatal neurologic illness in humans and horses, nearly 80% of diagnosed infections are asymptomatic [16]. In a 2004 study of patients with AFI in Haiti, post-Hurricane Jeanne, two of 116 (1.72%) patients screened were diagnosed as having had WNV infection, based on results of a microsphere-based immunoassay and a plaque reduction neutralization test [17].

##### ZIKV

ZIKV was isolated in Haiti as early as May 2014 from serum collected from schoolchildren presenting with AFI in the Gressier region of Haiti during the 2014 CHIKV epidemic; this was also the earliest documented ZIKV isolation in the hemisphere [12]. With the rapid spread of ZIKV in Brazil and its association with microcephaly in pregnant women, the Haiti Ministry of Health and Population (MSPP) implemented a national surveillance system to monitor cases of ZIKV infection in 2016 [18]. Such an initiative resulted in the detection of nearly 3000 cases with symptoms consistent with ZIKV infection, including 29 with suspected Zika-associated congenital microcephaly and 22 pregnant women [18]. Additional studies have confirmed local transmission of the virus in Haiti between 2014 and 2016 [12,19].

Although the MSPP has not officially reported ZIKV cases since 2016, two children presenting with AFI in the 2021 surveillance study described above were culture positive for ZIKV [13]. On phylogenetic analysis, the identified strains matched the first ZIKV strain identified in Haiti in 2014, consistent with the persistence of this strain in Haiti [13]. ZIKV was also isolated from *Aedes aegypti* mosquitoes, including, in one instance, from a mosquito pool consisting exclusively of male mosquitoes, consistent with transovarian transmission of the virus [20]. Phylogenetic studies indicated that ZIKV strains circulating in the Americas fell into three distinct clades [13,20]: isolates from clade 1 were identified in Brazil, including the Haiti 2021 ZIKV strain [13]; isolates from clade 2 were predominantly from Colombia, Panama, and Venezuela [20]; and isolates from clade 3 were from multiple locations in the Caribbean and the United States [20].

SPOV is temporarily phylogenetically classified by the International Committee for the Taxonomy of Viruses under the *Orthoflavivirus genus* but has not been approved as a distinct species [21]. SPOV and ZIKV are genetically closely related, and there is substantial cross-reactivity in serologic screening [22]. Contrary to ZIKV, little is known regarding the clinical and epidemiologic characteristics of SPOV infection [22]. Although SPOV infections are rarely associated with severe disease, a subset of patients has reported signs and symptoms suggestive of vascular leakage and neurological complications such as photophobia, vertigo, disorientation, and transient ocular paresis [22]. SPOV was isolated in Haiti from a pool of *Culex quinquefasciatus* mosquitoes collected in July 2016 in Haiti; to date, no human cases have been reported [10].

#### Alphaviruses

The genus *Alphavirus* consists primarily of mosquito-borne viruses, although other hematophagous insects, including ticks and lice, have been implicated in viral transmission [23]. This review identified five different alphaviruses for which there is evidence of animal or human infection in Haiti: CHIKV, North American Eastern equine encephalitis virus (EEEV)*,* Western equine encephalitis virus (WEEV), South American equine encephalitis virus (Madariaga virus, MADV)*,* and Mayaro virus (MAYV)*.*

##### CHIKV

The Asian clade of CHIKV was introduced into the Americas in December 2013, with the first cases identified on St. Martin in the Caribbean [24]. The virus subsequently spread rapidly through the Caribbean and into South America [25]. In April 2014, Haiti experienced a rapid spread of the infection [26]. Serologic studies documented the extensive spread of CHIKV throughout the country [27]. Cases of CHIKV infection among travelers returning from Haiti were also reported [28]. In a study of children with confirmed CHIKV infection, the most common symptoms were arthralgias (64% of cases), followed by myalgias (58%) and headache (39%) [19]. Arthralgias and myalgias were significantly more common among children infected with CHIKV as compared with children in the same cohort with confirmed DENV infection [19]. On phylogenetic analysis, Asian clade CHIKV strains within the initial epidemic peak were virtually identical to CHIKV Asian clade strains also isolated from *Aedes aegypti* mosquitoes but not from *Ae. albopictus* [29]*.*

In 2021, during a study of the etiology of AFI in schoolchildren in Fond Parisien, Haiti, CHIKV was isolated from two children, aged 3 and 4 years [13]. On phylogenetic analysis, these strains were within the East/Central/South African clade and, more specifically, the “American” subclade, which was previously identified in pools of *Ae. albopictus* mosquitoes in Haiti in 2016 (including a pool of male *Ae. albopictus*) [29]. In this study, 78% of children aged 7 years or older (those who would have been alive during the 2014 epidemic) were seropositive for CHIKV infection, compared with 2% of children under the age of 7 years [13].

##### North American EEEV

The North American variant of EEEV is endemic to eastern North America and the Caribbean [30]. To date, there is no evidence of EEEV circulation in humans in Haiti; however, during a serosurvey of non-migratory bird and bat species conducted in the southern peninsula of Haiti in 1972, neutralizing antibodies to EEEV were detected in a great blue heron [15].

##### WEEV

WEEV mainly circulates in the western regions of the United States, Canada, and South and Central America [31]. To date, no cases of WEEV infection in humans have been reported in Haiti. In the 1972 serosurvey referenced above, neutralizing antibodies to WEEV were reported in a fruit bat and a village weaver bird [15].

##### MADV

MADV is the South American variant of EEEV; on phylogenetic analysis, these strains constitute lineages II, III, and IV of what was previously designated as EEEV [32]. The virus appears to have been introduced to Haiti from Panama sometime between October 2012 and January 2015, based on phylogenetic and molecular clock data [33]. MADV was isolated in Haiti for the first time from eight children who presented with AFI to a school clinic between 2015 and 2016 [33]. When the symptoms of children with MADV infection were compared with those of children infected with other arboviruses, MADV symptoms most closely resembled those reported by children infected with DENV [33], with the frequency of arthralgias significantly reduced when compared with that in children with CHIKV infection [33].

##### MAYV

Reported symptoms of MAYV infection include fever with chills, headache, skin rash, and mild to severe and persistent arthralgia, with the majority of cases being reported in Brazil, Peru, and Ecuador [34]. MAYV has been isolated in Haiti in sera collected from five schoolchildren with AFI [35]. Phylogenetic/phylodynamic analysis of these strains suggests that there have been at least three independent introductions of MAYV into Haiti [35]. The rising number of reported cases and the expansion of the geographical range of MAYV far beyond the Amazon basin to other regions of South America and the Caribbean, including Haiti, represent a significant public health concern [[35], [36], [37], [38]]. Analyses of strains from the Caribbean and South America demonstrate recent recombination events and adaptation to a broad host and vector range, with a possible linkage to human population movements [38]. The spatial and directional spread of various MAYV strains coincided with the movement of Haitian migrants relocating to Brazil and other South American countries after the Haiti 2010 earthquake [38,39].

#### Orthobunyaviruses

*Orthobunyaviruses* are characterized by a tripartite, negative-sense ribonucleic acid genome; this genus contains various species and represents the largest genus within the Peribunyaviridae family [9]. Two orthobunyaviruses have been detected in Haiti, namely Melao virus (MELV) and Oropouche virus (OROV).

##### MELV

MELV was first isolated from *Aedes* and *Psorophora* spp. mosquitoes captured in the Melao forest in Trinidad [40]. There is limited documentation of MELV-associated human illnesses. The virus was isolated in cell culture from the plasma of five patients in Haiti in 2014 [7]. The most commonly reported clinical findings included fever, headache, abdominal pain, lymphadenopathy, neutropenia, and lymphocytosis [7]. Based on phylogenetic analysis, the Haitian MELV sequences clustered within a single clade together with an isolate reported from Trinidad and Tobago [7].

##### OROV

OROV is an important cause of AFI in South and Central America, with a recent increase in global incidence, including among US travelers [41]. OROV infection has symptoms similar to those of CHIKV, with 65% of patients in one study reporting arthralgias, together with headache and retro-orbital pain [41]. Rare neurological complications such as aseptic meningitis and cases with adverse pregnancy outcomes have also been reported among patients with OROV infection [41]. OROV ribonucleic acid was detected in the plasma of a child in Haiti in 2014 who had initially been diagnosed, based on clinical presentation, as having CHIKV infection [7]. Phylogenetic data suggest that the OROV strain identified in Haiti may be a divergent lineage related to, but distinct from, other previously identified genotype I sequences from Brazil [7]. Haiti has recently identified possible cases of OROV infection among young children, but no official information from authoritative or formal sources has been reported [42].

## Discussion

Twelve arboviruses from three distinct genera have been reported in Haiti between 1976 and 2025; all are transmitted by mosquitoes, except for OROV, which is primarily transmitted by biting midges. DENV is the most frequently reported arbovirus in Haiti, with all four serotypes having been reported to circulate in the country. It is surprising that no cases of DHF or DSS have been reported in this population. This may simply represent a failure to recognize or report these more severe forms of dengue. It has also been hypothesized, based on studies in Colombia that this reflects the existence of a protective gene among people of African descent [43]; these are potentially important observations, and further genetic studies to explore this hypothesis are clearly needed.

### Hypoendemicity of CHIKV and ZIKV in Haiti

Major outbreaks caused by CHIKV and ZIKV in Haiti are well documented, and even though reported case numbers are currently low, the recent isolation of both viruses from children with AFI suggests that both remain endemic (albeit at low levels) in Haiti [13]. Both ZIKV and CHIKV have been isolated from male mosquitoes, suggestive of transovarian transmission [20,29]. The genomic sequence of the 2021 ZIKV strain was nearly identical to the original 2014 isolate, consistent with the persistence of this particular strain within Haiti [13]. In contrast, the major 2014 CHIKV epidemic was associated with the Asian clade of the virus (which was only isolated from *Ae. aegypti*) [44], whereas the CHIKV detected in 2021 in *Ae. albopictus* was from the East/Central/South African clade [13]. Given the increasingly wide range of *Ae. albopictus* in the southern United States, these observations underscore the potential for further spread of this clade within the US [45].

### Circulation of emerging arboviral species within Haiti and among migrant populations

Documentation of MAYV, MADV, SPOV, MELV, and OROV infections is based on molecular studies and viral culture. While there is evidence of local transmission, these viruses do not appear to have been responsible for major outbreaks. As previously observed in malaria studies, which provide supportive evidence of the movement of *Plasmodium* species between Haiti and Jamaica through human migration [46,47], phylogenetic studies presented in this review support similar patterns for arboviral transmission across the Caribbean and South America [7,10,33,35]. There have also been recent reports highlighting the potential for high-altitude windborne transmission of mosquitoes carrying a variety of VBDs, which may also have contributed to the movement of viral strains among countries [48]. Currently available data on EEEV, SLEV, WEEV, and WNV circulation in Haiti are largely based on serologic assays, which may have had issues with cross-reactivity [15,17].

### Haiti’s sociopolitical and environmental landscapes and arboviral transmission

Haiti has the perfect conditions for VBD transmission. Lasting conflicts that limit public health surveillance activities and cause unrelenting mass displacements of families within and outside Haiti may facilitate disease dissemination locally and beyond the country’s borders. The ongoing United Nations-led Multinational Security Support mission in Haiti [49], which is assisting in neutralizing the gangs that control most of Haiti’s capital and surrounding cities, may facilitate the movement of new arbovirus species between Haiti and the countries from which personnel are drawn. As previously observed following Hurricane Jeanne in Haiti in 2004 [17], the aftermath of Hurricane Melissa, which devastated the Caribbean, including Haiti, in late October 2025, may trigger surges in VBDs, as seen in Cuba [50]. Without active public health surveillance in Haiti, it is difficult to fully assess such impacts. On the other hand, due to existing immunity against ZIKV and CHIKV, Haiti may be at reduced risk of another outbreak of these two viruses despite evidence supporting their hypoendemicity [13]. Furthermore, immunity to these viruses may confer some protection against future outbreaks of related, cross-reactive arbovirus species.

### Arboviral surveillance, progress, and challenges

Despite ongoing efforts by Haiti’s MSPP, through its Directorate of Epidemiology, Laboratories, and Research (DELR), in collaboration with the Centers for Disease Control and Prevention, the Pan American Health Organization, and other international partners to strengthen public health surveillance capacity, the country faces various shortcomings in monitoring and controlling various emerging and re-emerging infectious diseases [51,52]. Lasting armed conflicts, limited public health funding, and challenges in developing and retaining a qualified local public health workforce continue to hamper public health surveillance programs [53]. Collective efforts deployed over the last decades, particularly by the international research community, have resulted in the identification of various bunyaviruses as well as emerging alphaviruses that were not previously reported on the island [7,10,33,35,36]. The use of advanced molecular diagnostic methods in Haiti since the earthquake, with a significant increase observed from 2014 onward (Figure 2), has played an important role in the detection of these new arboviral species in Haiti. These findings also support ongoing circulation of unrecognized arboviruses and other pathogens of public health importance in Haiti and underscore the urgent need to enhance public health surveillance to monitor these viruses and implement appropriate countermeasures. In addition, these efforts have led to the development of an initial local genomic database providing a genomic footprint for various arboviral strains with their respective geographical linkage [7,33,35,36,44,54]. Such a database is crucial for understanding and monitoring regional and global viral transmission, viral evolution, and predicting future outbreaks.

### Immediate priorities and forward strategies

Serologic diagnostic methods should be used cautiously when assessing population immunity to existing or emerging arbovirus species in Haiti, particularly those belonging to the *Alphavirus*and *Orthoflavivirus* genera, due to the circulation of arboviruses that share similar antigenic epitopes, thus making them cross-reactive in serologic assays. Access to reliable, innovative diagnostic methods and phylogenetic analyses should be considered to enhance arboviral surveillance in Haiti. They are essential for accurate identification of arboviral species, tracking viral evolution and geographical spread, and increasing our understanding of arboviral transmission patterns within Haiti, across the Caribbean, and more broadly, the Americas. There is a critical need to promote local access to innovative and reliable diagnostic techniques in resource-limited settings like Haiti, where various pathogens of public health concern are endemic. These include virus culture, polymerase chain reaction, next-generation sequencing, and phylogenetic/phylodynamic analyses. They are essential for accurate identification of arboviral species, tracking viral evolution and geographical spread within Haiti, across the Caribbean, and more broadly, the Americas. Next-generation sequencing offers a crucial diagnostic alternative for identifying endemic and/or newly emerging pathogens in patients with AFI in Haiti. Alternatively, *in situ* detection devices, including those used to detect ZIKV [55] and MAYV [56], can be leveraged for both accurate and convenient arbovirus surveillance in Haiti.

### Limitations

Although this review summarizes current salient data regarding arbovirus transmission in Haiti, it has several limitations. First, the findings are restricted to the databases and studies selected based on the eligibility criteria. Furthermore, Haiti primarily relies on partners such as the Pan American Health Organization and the Centers for Disease Control and Prevention to monitor and report emerging and endemic infectious diseases. Future studies should expand the inclusion criteria and information sources to include these resources and other gray literature. Second, an important limitation to highlight is the variability across individual studies, including differences in the robustness of diagnostic methods used to identify these 12 arboviruses, sampling methods, sample sizes, study design, and settings. Half of the included studies were conducted in Haiti’s Ouest Department, whereas the remainder comprised three national surveys, 13 cases among returning travelers, and two studies outside the Ouest Department. As previously reported [57], this study underscores the significance of surveillance data from infected returning travelers for understanding the local prevalence of various infectious diseases in areas with limited public health surveillance. By the same token, given the historical and persistent challenges surrounding public health surveillance capacity in this Caribbean nation, we cannot rule out the unrecognized or underreported circulation of other emerging or re-emerging arboviruses.

## Conclusion

The increase in the geographical spread of many arboviruses in the Americas and the incidence of diseases associated with them are major public health concerns. This review provides a comprehensive overview of arboviruses that are or have been circulating in Haiti between 1976 and 2025. Haiti’s climate, socioeconomic conditions, limited healthcare infrastructure, and collapse of the public health surveillance system make it a potential hub for arbovirus transmission. Lasting conflict and continuous large-scale migration are increasing the risk of the spread of these pathogens within the country and potentially across the Caribbean region and the Americas. DENV is hyperendemic (with all four serotypes reported), yet cases of DHF and DSS have not been reported in Haiti. Future research is needed to investigate the incidence of ZIKV, DENV, and other arbovirus infections among Haitian pregnant women and to characterize any associated adverse maternal, fetal, and infant outcomes. Particularly, careful attention should be given to OROV following the report of molecular evidence of its circulation in Haiti, due to presumably low population immunity and the virus’s adverse impact on pregnancy.

## CRediT authorship contribution statement

**Rigan Louis:** Writing – review & editing, Writing – original draft, Methodology, Formal analysis, Data curation, Conceptualization. **Sk Nazmus Sakib:** Writing – review & editing, Formal analysis. **Z. Hugh Fan:** Writing – review & editing, Funding acquisition. **Leslie Ann Parker:** Writing – review & editing. **John A Lednicky:** Writing – review & editing. **John Glenn Morris Jr.:** Writing – review & editing, Supervision, Funding acquisition, Formal analysis, Conceptualization. **Valery Madsen Beau De Rochars:** Writing – review & editing.

## Declaration of competing interest

The authors have no competing interests to declare.

## Appendix Supplementary materials

Image, application 1

Image, application 2

Image, application 3
