# Supplementary material for: Emergence and endemicity of arboviruses in Haiti: a review
Source: IJID Reg. 2026 Jul 26;20:100964. doi: 10.1016/j.ijregi.2026.100964 (PMC13571743; doi:10.1016/j.ijregi.2026.100964)
Supplement: Supplementary file 2 [file mmc2.docx]

**Table S2**

Database search strategy

| **Database** | **Search Date** | **Search strategy with relevant key terms and MESH terms** | **Retrieved** |
| --- | --- | --- | --- |
| PubMed | August 2025 | ("Dengue"[MeSH Terms] OR "Dengue Virus"[MeSH Terms] OR "Dengue"[Title/Abstract] OR "Dengue Virus"[Title/Abstract] OR "DENV"[Title/Abstract] OR ("Zika Virus"[MeSH Terms] OR "zika"[Title/Abstract] OR "Zika Virus"[Title/Abstract] OR "ZIKV"[Title/Abstract] OR "spondweni"[Title/Abstract] OR "spondweni virus"[Title/Abstract] OR "SPONV"[Title/Abstract]) OR ("Chikungunya virus"[MeSH Terms] OR "chikungunya"[Title/Abstract] OR "Chikungunya virus"[Title/Abstract] OR "CHIKV"[Title/Abstract] OR "CHIKV"[Title/Abstract]) OR ("Yellow Fever"[MeSH Terms] OR "Yellow fever virus"[MeSH Terms] OR "Yellow Fever"[Title/Abstract] OR "YFV"[Title/Abstract]) OR ("West Nile Fever"[MeSH Terms] OR "West Nile virus"[MeSH Terms] OR "West Nile"[Title/Abstract] OR "West Nile virus"[Title/Abstract] OR "WNV"[Title/Abstract]) OR ("Japanese encephalitis"[Title/Abstract] OR "JEV"[Title/Abstract]) OR ("encephalitis, tick borne"[MeSH Terms] OR "tick-borne encephalitis"[Title/Abstract] OR "tick-borne encephalitis virus"[Title/Abstract] OR "TBEV"[Title/Abstract]) OR ("rift valley fever"[MeSH Terms] OR "Rift Valley fever virus"[MeSH Terms] OR "rift valley fever"[Title/Abstract] OR "RVFV"[Title/Abstract]) OR ("hemorrhagic fever, crimean"[MeSH Terms] OR "Crimean-Congo"[Title/Abstract] OR "CCHFV"[Title/Abstract]) OR ("Sindbis Virus"[MeSH Terms] OR "sindbis"[Title/Abstract]) OR ("Ross River virus"[MeSH Terms] OR "Ross River"[Title/Abstract] OR "RRV"[Title/Abstract]) OR "Barmah Forest"[Title/Abstract] OR ("mayaro"[Title/Abstract] OR "mayaro virus"[Title/Abstract] OR "MAYV"[Title/Abstract]) OR "oropouche"[Title/Abstract] OR ("st louis encephalitis"[Title/Abstract] OR "SLEV"[Title/Abstract]) OR ("Eastern equine encephalitis"[Title/Abstract] OR "EEEV"[Title/Abstract] OR "Madariaga"[Title/Abstract] OR "Madariaga virus"[Title/Abstract] OR "MADV"[Title/Abstract] OR ("Eastern equine encephalitis"[Title/Abstract] AND ("south america"[Title/Abstract] OR "South American"[Title/Abstract] OR "lineage"[Title/Abstract] OR "lineage II"[Title/Abstract] OR "lineage IIB"[Title/Abstract] OR "lineage III"[Title/Abstract]))) OR ("Western equine encephalitis"[Title/Abstract] OR "WEEV"[Title/Abstract]) OR ("Venezuelan equine encephalitis"[Title/Abstract] OR "VEEV"[Title/Abstract]) OR "powassan"[Title/Abstract] OR ("La Crosse virus"[MeSH Terms] OR "La Crosse encephalitis"[Title/Abstract]) OR "Jamestown Canyon"[Title/Abstract])  AND  ("Haiti"[MeSH Terms] OR "Haiti"[Title/Abstract]) | 112 |
