# Supplementary material for: Emergence and endemicity of arboviruses in Haiti: a review
Source: IJID Reg. 2026 Jul 26;20:100964. doi: 10.1016/j.ijregi.2026.100964 (PMC13571743; doi:10.1016/j.ijregi.2026.100964)
Supplement: Supplementary file 3 [file mmc3.docx]

**Table S3.** Characteristics of the included articles

| **Authors year of publication** | **Location^#^**  **Department/City** | **Reported arbovirus** | **Subject** | **Diagnostic**  **methods** |
| --- | --- | --- | --- | --- |
| Ventura, 1976 [1] | Ouest/PAP | DENV-2 | Haitian residents | Serologic/culture |
| MC Lean, 1979 [2] | Nippes/Miragoane | SLEV/EEEV/WEEV | Bird/Bat | PRNT |
| CDC/MMWR, 1994 [3] | UN personnel^##^ | DENV-1-2 | Travelers returning from Haiti | RT-PCR |
| Rossi et al., 1998 [4] | UN personnel | DENV-1-2-4 | Travelers returning from Haiti | Culture |
| Halstead et al., 2001 [5] | Ouest/PAP | DENV-1-to-4 | Haitian residents | PRNT |
| Beatty et al., 2007 [6] | Artibonite/Gonaives | DENV/WNV | Haitian residents | IgM/PRNT |
| Rioth et al., 2011 [7] | Ouest-PAP | DENV-1-to-4 | Haitian residents | PRNT |
| CDC/MMWR, 2011 [8] | UN personnel | DENV-1 | Travelers returning from Haiti | RT-PCR |
| Sharp et al., 2012 [9] | USA | DENV-1 | Travelers returning from Haiti | RT-PCR/Culture |
| Anderson et al.,2014 [10] | USA | CHIKV | Traveler returning from Haiti | RT-PCR/IgM |
| Burdino et al., 2015 [11] | Italy | CHIKV | Travelers returning from Haiti | Serologic |
| Serre et al., 2015 [12] | Spanish | DENV-4 | Travelers returning from Haiti | RT-PCR |
| Menard et al., 2015 [13] | France | DENV-4 | Haitian diaspora^###^ | RT-PCR/IgM/IgG |
| Poirier et al. ,2016 [14] | Ouest-Leogane | CHIKV | Haitian residents | IgG |
| ROSSINI et al., 2016 [15] | Italy | CHIKV | Travelers returning from Haiti | RT-PCR/IgM |
| Lednicky et al., 2016 [16] | Ouest/Gressier | DENV-1/ZIKV | Haitian residents | RT-PCR/culture |
| Lednicky et al., 2016 [17] | Ouest/Gressier | MAYV | Haitian residents | RT-PCR/Culture |
| Barzon et al., 2016 [18] | Italy | ZIKV | Traveler returning from Haiti | IgM/RT-PCR |
| Kassavetis et al., 2016 [19] | USA | ZIKV | Haitian diaspora | IgM/PRNT |
| Nicastri et al., 2016 [20] | Italy | ZIKV | Traveler returning from Haiti | RT-PCR |
| Whrite SK et al., 2017 [21] | Ouest/Gressier | CHIKV | Haitian residents | Culture |

| **Authors and**  **year of publication** | **Location^#^**  **Department/City** | **Reported arbovirus** | **Subject** | **Diagnostic**  **methods** |
| --- | --- | --- | --- | --- |
| Elbadry et al., 2017 [22] | Ouest/Gressier | DENV-4 | Haitian residents | RT-PCR/Culture |
| Weppelmann et al.,2017 [23] | Ouest/South-East | DENV/WNV | Haitian residents | IgG |
| Iovine et al., 2017 [24] | USA | DENV-2/ZIKV | Traveler returning from Haiti | RT-PCR/culture |
| Journel I et al., 2017 [25] | National | ZIKV | Haitian residents | RT-PCR |
| White et al., 2018 [26] | Ouest/Gressier | CHIKV | Mosquitoes | RT-PCR/culture |
| Rogier et al., 2018 [27] | National | CHIKV | Haitian residents | IgG |
| White et al., 2018 [28] | Ouest/Gressier | SPONV | Mosquitoes | RT-PCR |
| Ball et al., 2019 [29] | Ouest/Gressier | CHIKV/DENV1-2-4/  MAYV/ZIKV/ | Haitian residents | Culture/RT-PCR |
| Blohm et al., 2019 [30] | Ouest/Gressier | MAYV | Haitian residents | Culture/RT-PCR |
| Lednicky et al.,2019 [31] | Ouest/Gressier | MADV | Haitian residents | RT-PCR/culture |
| Elbadry et al., 2021 [32] | Ouest/Gressier | MELV/OROV | Haitian residents | Culture/RT-PCR |
| Chan et al., 2022 [33] | National | DENV-2/CHIKV | Haitian residents | Serologic |
| Alam et al., 2022 [34] | Ouest/Gressier | ZIKV | Haitian residents & Mosquitoes | Culture |
| Estep et al., 2024 [35] | Ouest/Leogane | DENV-1-2-3 | Mosquitoes | RT-PCR |
| Louis et al., 2025 [36] | Ouest/Fonds-Parisien | ZIKV/DENV-2/  CHIKV | Haitian residents | RT-PCR |

**Table S3.** Continued

^#^The country of origin is provided for travelers returning from Haiti.

^##^United Nation personnel returning from Haiti

^###^Haitian diaspora = People of Haiti origin living outside Haiti

**References**

[1]	Ventura AK. ENDEMIC DENGUE VIRUS-INFECTION IN HISPANIOLA .1. HAITI. JOURNAL OF INFECTIOUS DISEASES 1976;134:436–41. https://doi.org/10.1093/infdis/134.5.436.

[2]	MC LEAN R. PREVALENCE OF SELECTED ZOONOTIC DISEASES IN VERTEBRATES FROM HAITI, 1972. JOURNAL OF WILDLIFE DISEASES 1979;15:327–30.

[3]	CDC-MMWR. Dengue Fever Among U.S. Military Personnel — Haiti, September–November, 1994 1994.

[4]	Rossi C, Drabick J, Gambel J, Sun W, Lewis T, Henchal E. Laboratory diagnosis of acute dengue fever during the United Nations Mission in Haiti, 1995-1996. AMERICAN JOURNAL OF TROPICAL MEDICINE AND HYGIENE 1998;59:275–8. https://doi.org/10.4269/ajtmh.1998.59.275.

[5]	Halstead S, Streit T, Lafontant J, Putvatana R, Russell K, Sun W, et al. Haiti: Absence of dengue hemorrhagic fever despite hyperendemic dengue virus transmission. AMERICAN JOURNAL OF TROPICAL MEDICINE AND HYGIENE 2001;65:180–3. https://doi.org/10.4269/ajtmh.2001.65.180.

[6]	Beatty M, Hunsperger E, Long E, Schürch J, Jain S, Colindres R, et al. Mosquitoborne infections after Hurricane Jeanne, Haiti, 2004. EMERGING INFECTIOUS DISEASES 2007;13:308–10. https://doi.org/10.3201/eid1302.061132.

[7]	Rioth M, Beauharnais C, Noel F, Ikizler M, Mehta S, Zhu Y, et al. Serologic Imprint of Dengue Virus in Urban Haiti: Characterization of Humoral Immunity to Dengue in Infants and Young Children. AMERICAN JOURNAL OF TROPICAL MEDICINE AND HYGIENE 2011;84:630–6. https://doi.org/10.4269/ajtmh.2011.10-0323.

[8]	CDC-MMWR. Dengue virus infections among travelers returning from Haiti--Georgia and Nebraska, October 2010. MMWR Morb Mortal Wkly Rep 2011;60:914–7.

[9]	Sharp T, Pillai P, Hunsperger E, Santiago G, Anderson T, Vap T, et al. A Cluster of Dengue Cases in American Missionaries Returning from Haiti, 2010. AMERICAN JOURNAL OF TROPICAL MEDICINE AND HYGIENE 2012;86:16–22. https://doi.org/10.4269/ajtmh.2012.11-0427.

[10]	Anderson K, Pureza V, Walker P. Chikungunya: Acute Fever, Rash and Debilitating Arthralgias in a Returning Traveler From Haiti. JOURNAL OF TRAVEL MEDICINE 2014;21:418–20. https://doi.org/10.1111/jtm.12159.

[11]	Burdino E, Ruggiero T, Milia MG, Proietti A, Sergi G, Torta I, et al. Travelers with Chikungunya Virus Infection Returning to Northwest Italy from the Caribbean and Central America during June–November 2014. J Travel Med 2015;22:341–4. https://doi.org/10.1111/jtm.12219.

[12]	Serre N, Franco L, Sulleiro E, Rubio JM, Zarzuela F, Molero F, et al. Concurrent Infection with Dengue Type 4 and Plasmodium falciparum Acquired in Haiti. J Travel Med 2015;22:345–7. https://doi.org/10.1111/jtm.12222.

[13]	Menard A, Ninove L, Zandotti C, Leparc-Goffart I, Klitting R, Baronti C, et al. A secondary dengue 4 infection in a traveler returning from Haiti confirmed by virus isolation, complete genome sequencing and neutralisation assay: A brief report. TRAVEL MEDICINE AND INFECTIOUS DISEASE 2015;13:94–7. https://doi.org/10.1016/j.tmaid.2014.12.009.

[14]	Poirier MJ, Moss DM, Feeser KR, Streit TG, Chang GJ, Whitney M, et al. Measuring Haitian children’s exposure to chikungunya, dengue and malaria. Bull World Health Organ 2016;94:817-825A. https://doi.org/10.2471/BLT.16.173252.

[15]	ROSSINI G, GAIBANI P, VOCALE C, FINARELLI AC, LANDINI MP. Increased number of cases of Chikungunya virus (CHIKV) infection imported from the Caribbean and Central America to northern Italy, 2014. Epidemiology & Infection 2016;144:1912–6. https://doi.org/10.1017/S0950268815002940.

[16]	Lednicky J, De Rochars V, El Badry M, Loeb J, Telisma T, Chavannes S, et al. Zika Virus Outbreak in Haiti in 2014: Molecular and Clinical Data. PLOS NEGLECTED TROPICAL DISEASES 2016;10. https://doi.org/10.1371/journal.pntd.0004687.

[17]	Lednicky J. Mayaro Virus in Child with Acute Febrile Illness, Haiti, 2015. Emerg Infect Dis 2016;22:2000–2. https://doi.org/10.3201/eid2211.161015.

[18]	Barzon L, Pacenti M, Franchin E, Lavezzo E, Trevisan M, Sgarabotto D, et al. Infection dynamics in a traveller with persistent shedding of Zika virus RNA in semen for six months after returning from Haiti to Italy, January 2016. EUROSURVEILLANCE 2016;21:2–5. https://doi.org/10.2807/1560-7917.ES.2016.21.32.30316.

[19]	Kassavetis P, Joseph JM, Francois R, Perloff MD, Berkowitz AL. Zika virus-associated Guillain-Barré syndrome variant in Haiti. Neurology 2016;87:336–7. https://doi.org/10.1212/WNL.0000000000002759.

[20]	Nicastri E, Castilletti C, Liuzzi G, Iannetta M, Capobianchi MR, Ippolito G. Persistent detection of Zika virus RNA in semen for six months after symptom onset in a traveller returning from Haiti to Italy, February 2016. Eurosurveillance 2016;21. https://doi.org/10.2807/1560-7917.ES.2016.21.32.30314.

[21]	White SK, Morris JG Jr, Elbadry MA, Beau De Rochars VM, Okech BA, Lednicky JA. Complete Genome Sequences of Chikungunya Viruses Isolated from Plasma Specimens Collected from Haitians in 2014. Genome Announc 2017;5. https://doi.org/10.1128/genomeA.00148-17.

[22]	Elbadry MA, White SK, Loeb JC, Tagliamonte MS, Salemi M, Beau De Rochars VM, et al. Complete Genomic Sequence of Dengue Virus Serotype 4 Isolated from Plasma Collected from a Haitian Child in 2014. Genome Announc 2017;5. https://doi.org/10.1128/genomeA.01160-17.

[23]	Weppelmann T, Burne A, von Fricken M, Elbadry M, De Rochars M, Boncy J, et al. A Tale of Two Flaviviruses: A Seroepidemiological Study of Dengue Virus and West Nile Virus Transmission in the Ouest and Sud-Est Departments of Haiti. AMERICAN JOURNAL OF TROPICAL MEDICINE AND HYGIENE 2017;96:135–40. https://doi.org/10.4269/ajtmh.16-0422.

[24]	Iovine N, Lednicky J, Cherabuddi K, Crooke H, White S, Loeb J, et al. Coinfection With Zika and Dengue-2 Viruses in a Traveler Returning From Haiti, 2016: Clinical Presentation and Genetic Analysis. CLINICAL INFECTIOUS DISEASES 2017;64:72–5. https://doi.org/10.1093/cid/ciw667.

[25]	Journel I, Andrécy LL, Metellus D, Pierre JS, Faublas RM, Juin S, et al. Transmission of Zika Virus - Haiti, October 12, 2015-September 10, 2016. MMWR Morb Mortal Wkly Rep 2017;66:172–6. https://doi.org/10.15585/mmwr.mm6606a4.

[26]	White S, Mavian C, Salemi M, Morris J, Elbadry M, Okech B, et al. A new “American” subgroup of African-lineage Chikungunya virus detected in and isolated from mosquitoes collected in Haiti, 2016. PLOS ONE 2018.

[27]	Rogier EW, Moss DM, Mace KE, Chang M, Jean SE, Bullard SM, et al. Use of Bead-Based Serologic Assay to Evaluate Chikungunya Virus Epidemic, Haiti. Emerg Infect Dis 2018;24:995–1001. https://doi.org/10.3201/eid2406.171447.

[28]	White S, Lednicky J, Okech B, Morris J, Dunford J. Spondweni Virus in Field-Caught Culex quinquefasciatus Mosquitoes, Haiti, 2016. EMERGING INFECTIOUS DISEASES 2018;24:1765–7. https://doi.org/10.3201/eid2409.171957.

[29]	Ball J, Elbadry M, Telisma T, White S, Chavannes S, Anilis M, et al. Clinical and Epidemiologic Patterns of Chikungunya Virus Infection and Coincident Arboviral Disease in a School Cohort in Haiti, 2014-2015. CLINICAL INFECTIOUS DISEASES 2019;68:919–26. https://doi.org/10.1093/cid/ciy582.

[30]	Blohm G, Elbadry M, Mavian C, Stephenson C, Loeb J, White S, et al. Mayaro as a Caribbean traveler: Evidence for multiple introductions and transmission of the virus into Haiti. INTERNATIONAL JOURNAL OF INFECTIOUS DISEASES 2019;87:151–3. https://doi.org/10.1016/j.ijid.2019.07.031.

[31]	Lednicky JA, White SK, Mavian CN, El Badry MA, Telisma T, Salemi M, et al. Emergence of Madariaga virus as a cause of acute febrile illness in children, Haiti, 2015-2016. PLoS Negl Trop Dis 2019;13:e0006972. https://doi.org/10.1371/journal.pntd.0006972.

[32]	Elbadry M, Duraes-Carvalho R, Blohm G, Stephenson C, Loeb J, White S, et al. Orthobunyaviruses in the Caribbean: Melao and Oropouche virus infections in school children in Haiti in 2014. PLOS NEGLECTED TROPICAL DISEASES 2021;15. https://doi.org/10.1371/journal.pntd.0009494.

[33]	Chan Y, Martin D, Mace KE, Jean SE, Stresman G, Drakeley C, et al. Multiplex Serology for Measurement of IgG Antibodies Against Eleven Infectious Diseases in a National Serosurvey: Haiti 2014–2015. Front Public Health 2022;10:897013. https://doi.org/10.3389/fpubh.2022.897013.

[34]	Alam M, Mavian C, Okech B, White S, Stephenson C, Elbadry M, et al. Analysis of Zika Virus Sequence Data Associated with a School Cohort in Haiti. AMERICAN JOURNAL OF TROPICAL MEDICINE AND HYGIENE 2022;107:873–80. https://doi.org/10.4269/ajtmh.22-0204.

[35]	Estep A, Sanscrainte N, Okech B. AEDES AEGYPTI KNOCKDOWN RESISTANCE MUTATIONS AND DENGUE VIRUS INFECTION IN HAITI. JOURNAL OF THE AMERICAN MOSQUITO CONTROL ASSOCIATION 2024;40:102–8.

[36]	Louis R, Moquin TL, Mavian C, Barthelemy A, Pu R, Anderson B, et al. Dengue and Other Arbovirus Infections among Schoolchildren, Haiti, 2021. Emerg Infect Dis 2025;31. https://doi.org/10.3201/eid3102.240791.
